# Autoimmunity and allergy control in adults submitted to complete thymectomy early in infancy

**DOI:** 10.1371/journal.pone.0180385

**Published:** 2017-07-07

**Authors:** Susana L. Silva, Adriana Albuquerque, Andreia J. Amaral, Quan-Zhen Li, Catarina Mota, Rémi Cheynier, Rui M. M. Victorino, M. Conceição Pereira-Santos, Ana E. Sousa

**Affiliations:** 1 Instituto de Medicina Molecular, Faculdade de Medicina, Universidade de Lisboa. Lisboa, Portugal; 2 Clinica Universitária de Imunoalergologia, Hospital de Santa Maria, Centro Hospitalar Lisboa Norte. Lisboa, Portugal; 3 Microarray Core Facility, University of Texas Southwestern Medical Center, Dallas, United States of America; 4 Clinica Universitária de Medicina 2, Hospital de Santa Maria, Centro Hospitalar Lisboa Norte. Lisboa, Portugal; 5 Cytokines and Viral Infections, Immunology Infection and Inflammation department, Institut Cochin, INSERM, U1016, Paris, France; 6 Centre National de la Recherche Scientifique, UMR8104, Paris, France; 7 Université Paris Descartes, Paris, France; Universite Paris-Sud, FRANCE

## Abstract

The contribution of the decline in thymic activity for the emergence of autoimmunity is still debatable. Immune-competent adults submitted to complete thymectomy early in life provide a unique model to address this question. We applied here strict criteria to identify adults lacking thymic activity based on sjTREC levels, to exclude thymic rebound and/or ectopic thymuses. In agreement, they featured severe naïve CD4 T-cell depletion and contraction of T-cell receptor diversity. Notwithstanding this, there was neither increased incidence of autoimmune disease in comparison with age-matched controls nor significant changes in their IgG/IgA/IgM/IgE autoreactivity profiles, as assessed through extensive arrays. We reasoned that the observed relative preservation of the regulatory T-cell compartment, including maintenance of naïve regulatory CD4 T-cells, may contribute to limit the emergence of autoimmunity upon thymectomy. Our findings have implications in other clinical settings with impaired thymic activity, and are particularly relevant to studies of autoimmunity in ageing.

## Introduction

The thymus is essential to the establishment of the “peripheral” T-cell compartment before birth and during the accelerated somatic growth of childhood, and contributes to its continuous replenishment until at least the sixth decade of life[1]. Thymus removal early in infancy during corrective cardiac surgery is, therefore, associated with marked contraction of the naïve T-cell subset, as well as with the presence of markers of premature immune senescence, as a result of homeostatic naïve T-cell proliferation/differentiation[1, 2]. This is thought to occur mainly in response to self and environmental antigens[2], raising the question whether early thymectomy leads to an increased risk of autoimmunity and/or allergic disease. The few studies available are not conclusive[3–8]. The discrepant results may be in part due to cohort heterogeneity regarding age, length of follow-up post-thymectomy and degree of residual thymic activity[3–8]. Notably, thymic recovery has been reported in some individuals [9, 10].

Autoimmunity and allergy are controlled by a subset of regulatory CD4 T-cells (Tregs), defined by FoxP3 expression[11–13]. Tregs generated in the thymus are particularly implicated in the maintenance of self-tolerance, since they are thought to have a more autoreactive TCR repertoire[14]. They egress from the thymus with a naïve phenotype (naïve-Tregs), and continuously replenish the fully-suppressor memory-Treg compartment throughout life.

We recently reported that naïve-Tregs are preserved in adults more than 18y (median 21y) after complete thymectomy early in infancy, despite the marked contraction of conventional naïve CD4 T-cells[15, 16]. Importantly, in contrast to other studies[3, 4], we specifically excluded individuals with evidence of remaining thymic activity, based on single-joint T-cell receptor excision circle (sjTREC) quantification[15, 16]. sjTRECs are by-products of TCR rearrangements during T-cell development in the thymus that are enriched in recent-thymic emigrants and progressively lost as cells divide in the periphery[17]. We strictly selected individuals with circulating levels of sjTREC/μl clearly below the lower level found in healthy subjects, in addition to a surgical report of complete thymus removal[15, 16]. In agreement with our data, naïve-Treg preservation was also found in a cohort of recently thymectomized children[18].

Here we investigated the possibility that the maintenance of naïve-Tregs limits the development of autoimmunity and/or allergy likely associated with the skewed conventional T-cell repertoire upon complete thymectomy[16].

## Patients and methods

We compared our cohort of adults with strictly defined complete thymus removal in early infancy with age-matched healthy individuals (Table 1). Thymectomized individuals were selected based on severely reduced sjTRECs/μl[15, 16] at the time of our evaluation (July 2011—October 2012).

**Table 1:** Clinical, epidemiologic and immunologic characterization.

		Age/Gender	Autoimmunity	Allergy	Atopy Phadiatop^®^^a^	ImmunoCAP ISAC^®^^b^	sjTRECs	CD8T-cells/μl	% naïve in CD8^c^	CD4T-cells/μl	% naïve in CD4^c^	% FoxP3^+^ in CD4	FoxP3^+^ CD4T-cells/μl	% CD39^+^ in mem Treg^d^	CTLA4 MFI inmem Treg^d^
Thymectomized																
Individual Code (Fig 1)	T1	22/F	No	No	-	-	0.52	367	7.3	799	13.0	3.1	21.8	21.7	879
	T2	24/M	No	Rhinitis	+	+^e^	0.05	239	25.8	654	48.8	5.3	33.7	79.2	1327
	T3	22/F	No	No	-	-	1.76	567	13.8	1153	15.3	5.6	43.2	76.4	1005
	T4	22/M	No	No	-	-	0.71	186	23.6	406	22.1	4.7	10.6	28.0	1229
	T5	26/M	No	No	-	-	0.45	366	4.2	1000	17.2	5.0	46.7	88.4	970
	T6	27/M	No	No	-	-	0.67	386	9.8	407	9.3	3.1	13.2	79.1	1346
	T7	26/M	No	Rhinitis	+	+^e^	0.05	352	4.8	785	10.3	5.1	37.5	90.6	1189
Cohort (n = 7)		24 (22–27)	0	2	2	2	0.52 ***	366 *	9.8 ***	785	15.3 **	4.9% *	27.8	77.8%	1189
		2F/5M					(0.05–1.8)	(186–567)	(4.2–25.8)	(406–1153)	(9.3–48.8)	(3.1–5.6)	(4.8–46.7)	(21.7–90.6)	(879–13461)
Controls																
Included in arrays (n = 7)		23 (21–25)	0	2^f^	1^f^	2^f^	15.8	501	44.1	942	40.0	2.9%	23.5	75.1%	1247
		3F/4M					(8.7–34.6)	(307–863)	(31.5–56.1)	(588–1192)	(34.9–46.6)	(1.6–5.4)	(11.8–51.6)	(34.4–80.6)	(808–1831)
Total (n = 20)		21 (18–29)	0	7^g^	n.a.	n.a.	17.2	583	48.3	967	42.2	2.9%	22.4	76.5%	1197
		12F/8M					(4.01–39.3)	(307–921)	(22.9–70.6)	(566–1315)	(29.2–57.7)	(1.2–5.4)	(9.2–51.1)	(34.4–84.7)	(808–1950)

n.a. Not applicable; F—female; M—male; Results are shown as median and range in brackets; Statistical analysis was performed with Graph Prism Version 5.01, using unpaired T-test or Mann-Whitney as appropriate

*, **,*** p value <0.05; 0.01; 0.001 respectively, in comparison with controls (n = 20). Thymectomized individuals are identified by individual code (T)

^a^ ImmunoCAP Phadiatop® (TermoFischer scientific, Waltham, MA) was performed according to manufacturer´s instructions. Results were expressed as positive or negative regarding the presence of IgE antibodies in the serum to a balanced mixture of relevant environmental allergens, indicating the patient is atopic/non-atopic, respectively.

^b^ ImmunoCAP ISAC® (TermoFischer scientific) results are evaluated using Phadia Microarray Image Analysis (MIA) software, on a semiquantitative basis; IgE values are presented in arbitrary units called ISAC standardized units (from 0.3 to 100 ISU); Values of >0.3 ISU were considered positive.

^c^ Naïve cells were defined as CCR7^+^RO^−^.

^d^ mem Treg (memory Treg) were defined as CD4^+^RO^+^FoxP3^+^.

^e^ ISAC^®^ detectable specific IgE (KU/l)–T2: rDer f2: 1.4; rDer p2: 2.2; rLep d2: 12; rOle e1: 2.2; T7: rBlo t5: 2.1; nDer f1: 14; rDer f2: 22; nDer p1: 19; rDer p2: 34.

^f^ Allergic manifestations, Phadiatop^®^, ISAC^®^ detectable specific IgE (KU/l) in the controls (C) included in the arrays- C3: rhinitis, Phadiatop^®^ +, rOlee1: 6.8; nPhp4: 3; rAlta1:5.3nDerf1 4.3; rDerf2 7.1; nDerp1:11; rDerp2 11; C6: peach allergy, Phadiatop^®^ –, rPru p3: 22; nJug r3: 4.

^g^ Allergic manifestations in controls: rhinitis (n = 4), rhinitis and asthma (n = 1), atopic dermatitis and rhinitis (n = 1); peach allergy (n = 1).

All subjects gave written informed consent for blood sampling. The study was approved by the Ethical Boards of Faculdade de Medicina da Universidade de Lisboa, Centro Hospitalar Lisboa Norte, and Hospital de Santa Cruz, Portugal.

An extensive structured questionnaire was applied to all individuals by the same investigator to assess clinical manifestations of autoimmunity and allergy, in parallel with the evaluation of serum reactivity to large panels of autoantigens and allergen components. Modules of ISAAC Questionnaire validated for Portuguese speaking adults were included in this evaluation[19]. Regarding autoimmunity, we applied an in-house developed detailed clinical questionnaire collecting the previous diagnosis and the signs/symptoms suggestive of autoimmune disease.

Ex-vivo phenotypic analysis of lymphocyte subsets was performed in freshly-collected whole blood by 8-color flow cytometry, as previously described[15, 16].

Autoantigen microarrays were used to detect IgG/IgM or IgA/IgE autoantibodies in serum to a panel of 125 autoantigens[20]. The same chip was used to test all individuals for each isotype. Serum samples were treated with DNAse I (1:50) before incubation with autoantigen arrays. Autoantibodies were detected in parallel arrays, with Cy3-labelled anti-human IgG and Cy5-labelled anti-human IgM (Jackson ImmunoResearch, West Grove, PA, USA), or Cy3-labeled anti-human IgA (Jackson ImmunoResearch) and TRITC-conjugated anti-human IgE (ThermoFisher Scientific) for imaging. Fluorescence was quantified using GenePix 4400A scanner with appropriate laser wavelengths and generated Tiff images. Genepix Pro 7.0 software was used to analyse the image and create the Gene Pix Results (GPR) file. The net fluorescence intensities were normalized using purified human IgG/IgM/IgA/gE spotted onto each array. Data obtained from duplicate spots were averaged. Signal-to-noise ratio (SNR) was used as a quantitative measure of the ability to resolve true signal from background noise, with SNR≥3 considered true signal. Normalized profiles of antigen-microarrays were log2 transformed, and gplots package[21]were used to generate non-supervised hierarchical clustering heatmaps of the Euclidean distance matrix estimated from serum autoantibody reactivities. R statistical environment[22] and Limma package[23] were used to test and infer for differential antigen reactivity.

In order to expose a putative increase in the frequency of IgE sensitizations (sIgE) upon complete thymectomy, we used ImmunoCAP ISAC® (TermoFischer scientific), a miniaturized immunoassay platform, to assess serum specific IgE (sIgE) to 112 allergen components from 51 aeroallergen or food sources. This test was performed in the same day for all individuals (Table 1), following the manufacturer´s instructions.

## Results

Adults with complete thymectomy early in infancy featured very low naïve CD4 and CD8 T-cell counts (Table 1), as expected[4, 5, 7, 8, 24–26]. Importantly, their Treg compartment was preserved, with evidence of similar expression levels of markers associated with suppressive function phenotype within memory-Tregs, based on FoxP3, CTLA-4 and CD39 expression levels [27, 28](Table 1).

**Fig 1:**
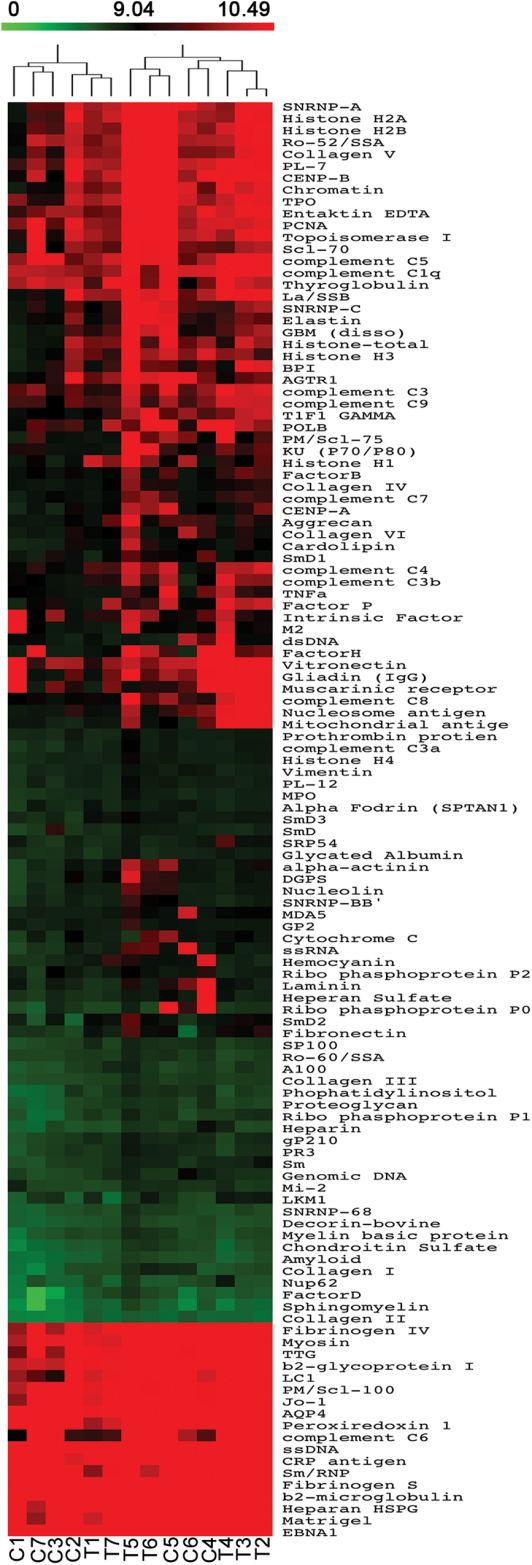
IgG autoantibody profiles. Heatmap of serum IgG autoantibody reactivity in adults thymectomized early in infancy (T) and controls (C) clustered by autoantigen and subject group. Reactivity intensity was normalized and log2-transformed, and 121 autoantibodies meeting minimal net fluorescence intensity requirements are presented. Key color bar corresponds to quantified reactivity.

**Fig 2:**
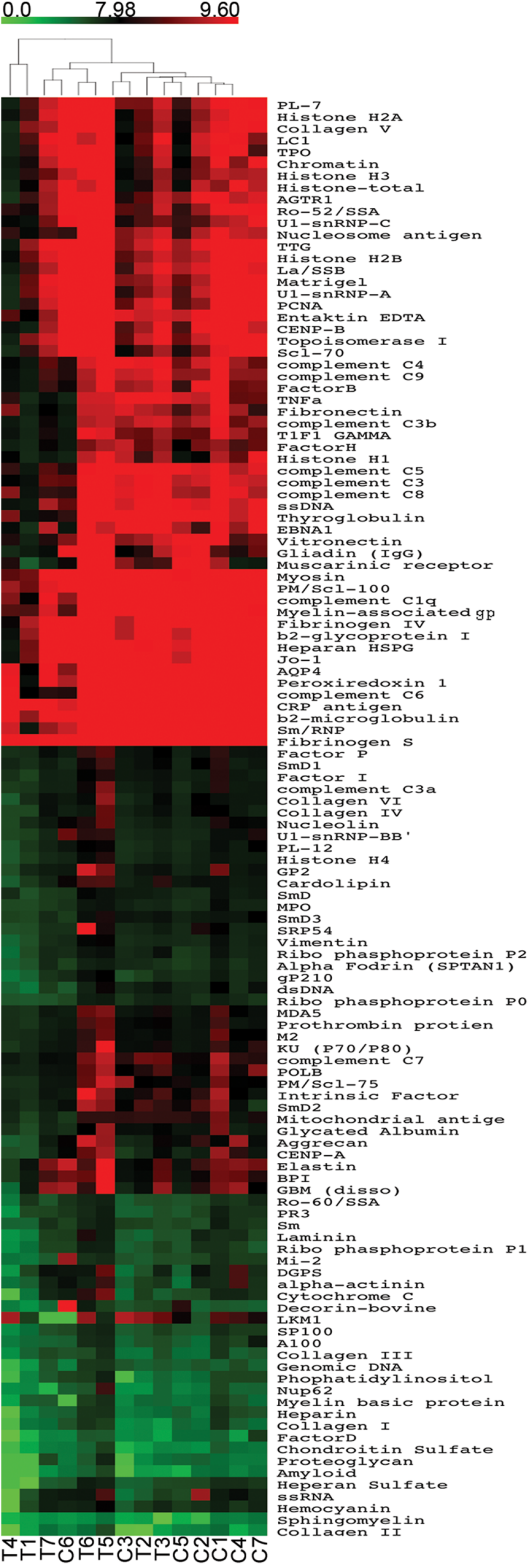
IgA autoantibody profile. Heatmaps of serum IgA autoantibody reactivity in adults thymectomized early in infancy (T) and controls (C), clustered by autoantigen and subject group. Reactivity intensities were normalized and log2-transformed; and 121 IgA autoantibodies meeting minimal net fluorescence intensity requirements are presented. Key color bar corresponds to quantified reactivity.

**Fig 3:**
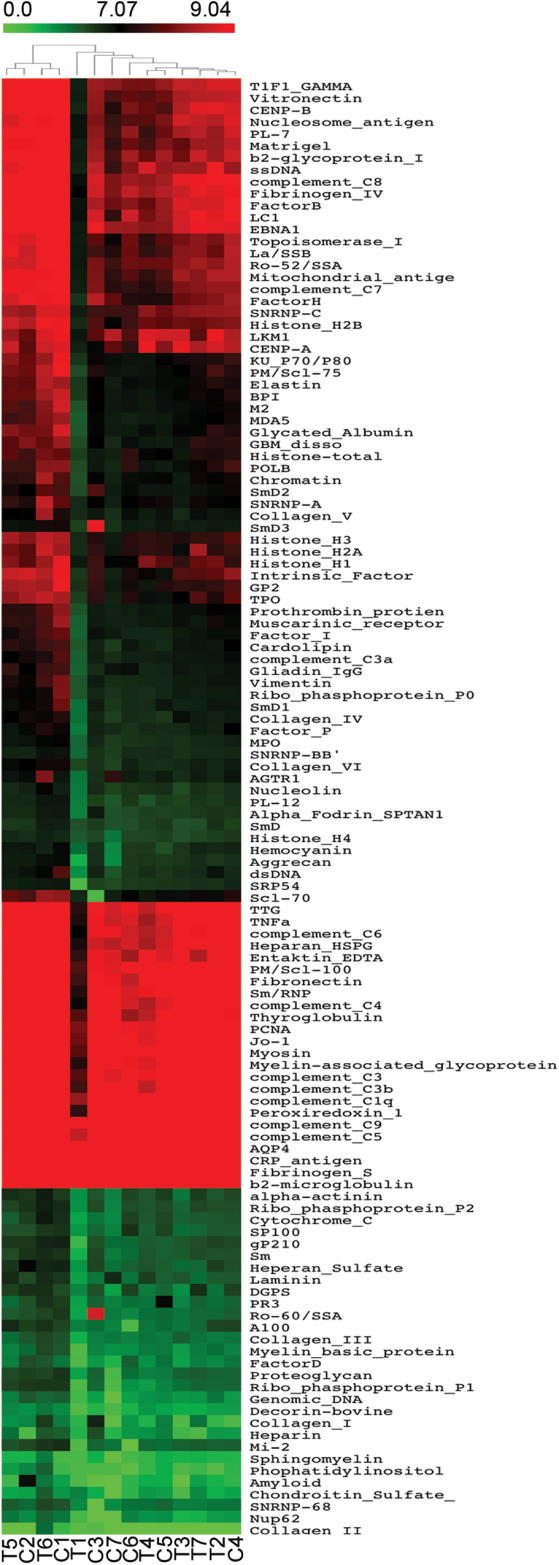
IgM autoantibody profile. Heatmaps of serum IgM autoantibody reactivity in adults thymectomized early in infancy (T) and controls (C), clustered by autoantigen and subject group. Reactivity intensities were normalized and log2-transformed; and 122 IgM autoantibodies meeting minimal net fluorescence intensity requirements are presented. Key color bar corresponds to quantified reactivity.

**Fig 4:**
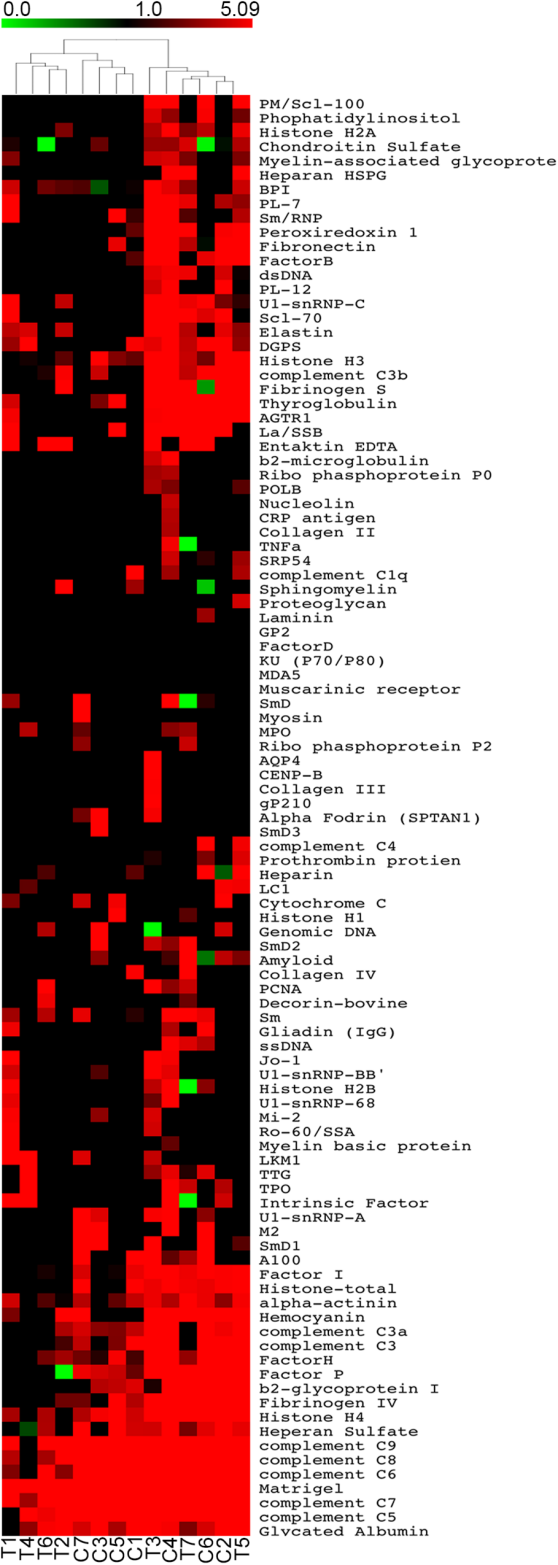
IgE autoantibody profiles. Heatmaps of serum IgE autoantibody reactivities in adults thymectomized early in infancy (T) and controls (C) clustered by autoantigen and subject group. Reactivity intensities were normalized and log2-transformed, and 100 IgE autoantibodies meeting minimal net fluorescence intensity requirements are presented. Key color bars correspond to quantified reactivity.

We found no autoimmune manifestations based on a structured clinical questionnaire. In order to reveal a possible subclinical increase in IgG, IgM, IgA and/or IgE autoreactivity, we took advantage of autoantigen array-based technology[29], an emergent approach with high sensitivity for early and large-scale autoantibody identification[29]. The autoreactivity profiles of the complete thymectomized adults were compared with age-matched representative controls (Table 1). By fitting a linear model to the normalized data (S1–S4 Tables), using R statistical environment[22] and Limma package[23], we did not find significant differences in serum IgG (Fig 1), IgA (Fig 2), IgM (Fig 3), as well as IgE (Fig 4) reactivity between cohorts (*P*>0.05). Moreover, the clustering analysis showed a clear inability to correctly assign thymectomized and controls on the basis of antigen reactivity profiles (Figs 1–4).

In spite of the lack of significant differences after applying the multiple test correction, when analysing IgG reactivity to distinct autoantigens, we identified 18 autoantibodies with higher expression within complete thymectomized than in age-matched healthy subjects, (S1 Table; *P*<0.05, before multiple testing correction). Their targets included nuclear antigens (Sp100, chromatin, histone H1, ribophosphoprotein P1, SmD2, SmD3, TIF1gamma, Sm), cytoplasmic/membrane proteins (mitochondrial antigen, tissue transglutaminase, liver cytosolic 1 antigen, sphingomyelin), cell matrix proteins (fibronectin), glomeruli-specific proteins (glomerular basement membrane) and circulating proteins (complement C3a, complement C4, complement C8, factor D). Interestingly, these autoantigens have been identified in systemic lupus erythematosus (SLE) patients[29], some of them with high specificity, such as SmD proteins. The SLE studies revealed an association between particular IgG autoantibody clusters and overall disease activity or lupus nephritis[29], one of which was partially reproduced in our complete thymectomized cohort (glomerular basement membrane/chromatin antibodies). Additionally, a cluster combining IgG autoantibodies to liver cytosolic antigen 1/mitochondrial antigen/tissue transglutaminase, antigens clearly associated with autoimmune liver disease[30], was also found in our thymectomized adults.

Recent data recall attention for a role of IgE reactivity against self-peptides in autoimmunity although their significance is still debatable[31]. We were able to identify IgE reactivity to a relatively small number of antigens in both cohorts (a median of 34 per individual in complete thymectomized versus 31 in controls in a total of 125 antigens included in the array, *P*>0.05). There was no statistical difference between healthy and thymectomized. We also found 4 IgE autoantibodies, not previously reported[31], for which we observed different levels of reactivity between thymectomized and control individuals.(heperan sulfate, Factor P, mitochondrial antigen M2, entaktin EDTA (S4 Table; *P*<0.05, before multiple testing correction), but in contrast to IgG, these differences did not feature a consistent trend between the two cohorts. These findings reinforce the need for further investigation on IgE autoreactivity[31].

Regarding allergy, we found no significant differences in the prevalence of clinical manifestations in complete thymectomyzed and age-matched control individuals (Table 1). Allergic rhinitis was the most frequent disease in both cohorts, as expected in young adulthood, and atopy was documented only in individuals with rhinitis (Table 1). We also performed a microarray to 112 allergen components that revealed positive sIgE in patients with allergic disease, in agreement with their clinical manifestations (Table 1). Our findings suggest that thymectomy in early age has no major impact on the degree of IgE-sensitization against food or airborne allergens.

## Discussion

Our study, that was strictly designed to include only adults without evidence of thymic activity upon thymus removal in infancy, revealed no increase of clinical/subclinical manifestations of allergy or of autoimmune diseases, as compared with a group of age matched healthy individuals. This occurred despite these individuals featured marked contraction of the conventional naïve T-cell pool and a restricted T cell-receptor diversity[16]. It is likely that the absence of autoimmune manifestations may be related to the parallel preservation of the Treg compartment, given the major role of regulatory T-cells in their control. Notably, the thymus-committed naïve-Treg population was found to be maintained in these adults by peripheral homeostatic mechanisms, despite the lack of thymic output since early in infancy[15]. Naïve-Tregs are considered to play a major role in the control of self-reactivity due to their enrichment in self-reactive T-cell receptors[14].

Nevertheless, beyond this well acknowledged role of the Treg compartment in the containment of autoimmunity, other factors may control the emergence of autoimmunity [32]. Autoimmune diseases are reported in 7.6–9.4% in the general population[33], and the relative contribution of constriction of the TCR repertoire is not well established [34], However, although we recognize the need for studies including a larger number of individuals, we believe that our findings significantly contribute to the field because we gathered a homogenous population of young adults without thymic activity and took advantage of highly sensitive methodologies to reveal possible subclinical increases in autoreactivity in thymectomized individuals.

Cohorts of adults submitted to thymectomy in infancy are expanding due to generalization of the access to early corrective/palliative cardiac surgery, though the eldest individuals are still in the fourth decade of life[1]. Therefore, both extended follow-up and larger cohorts will be very important. Although we found no significant differences between cohorts, the thymectomized group featured a trend to increased subclinical IgG autoreactivity against antigens with relevant clinical associations. Patients´ follow-up will ultimately reveal whether the relatively preserved Treg compartment will be able to limit overt progression towards autoimmune disease[29].

There are several previous studies addressing whether thymectomy performed early in life is associated with the subsequent development of autoimmunity and allergy[3–8]. An increase in the frequency of allergic manifestations was suggested based on questionnaires, although no data on skin testes or specific IgE quantifications were provided[3, 4, 7]. Conversely, there are no reports of autoimmune diseases based on clinical questionnaires [4–8]. Three of these studies also evaluated autoantibodies [3] [5, 7]. Two were performed in children without stratification according to thymic activity and found no increase in antibody titers against thyroglobulin, parietal cells and pancreatic islet cells[7], or rheumatoid factor and ANA [5]. The third included both children and adults grouped according to sjTREC levels and evidence of residual thymic tissue in subsequent surgeries or radiologic imaging, and reported an increase in the levels of anti-dsDNA without association with clinical symptoms or presence or absence of residual thymic tissue, although these individuals featured reduced sjTRECs[3].

Our study applied strict criteria to exclude individuals with remaining thymic activity due to incomplete thymus removal and/or ectopic thymus. Of note, cardiothoracic surgeries have revealed a high frequency of ectopic thymic tissue [35]. Moreover, we focused on adults with more than 18 years of follow-up post complete thymectomy, aiming a population homogeneity that we believe minimizes the limitation imposed by the relatively small numbers. Additionally, we used an innovative evaluation of subclinical autoreactivity/IgE-sensitizations in order to reveal possible immune dysregulation in individuals lacking thymic activity. It has been demonstrated autoantigen arrays correlate very well with data generated by ELISA with much higher sensitivity[29].

Individuals submitted to thymectomy in infancy have been considered a privileged model to address the contribution of the thymus and of peripheral mechanisms to control autoimmunity and allergy. Our data are consistent with the possibility that in the absence of thymic activity, peripheral tolerance limits the emergence of autoimmunity possibly through relative Treg preservation. These findings have implications for other clinical settings with impaired thymic activity. Particularly, they may steer the development of new strategies targeting peripheral immunoregulatory systems and thymus regeneration to control autoimmunity in the context of ageing or disease-associated thymus involution.

## Supporting information

S1 Table Normalized Log 2 transformed values of serum IgG autoreactivity intensity. Samples and antigens are in the same hierarchical order as shown in the corresponding heatmap figure.
(XLS)

S2 Table Normalized Log 2 transformed values of serum IgA autoreactivity intensity. Samples and antigens are in the same hierarchical order as shown in the corresponding heatmap figure.
(XLS)

S3 Table Normalized Log 2 transformed values of serum IgM autoreactivity intensity. Samples and antigens are in the same hierarchical order as shown in the corresponding heatmap figure.
(XLS)

S4 Table Normalized Log 2 transformed values of serum IgE autoreactivity intensity. Samples and antigens are in the same hierarchical order as shown in the corresponding heatmap figure.
(XLS)
